# Transcriptional Factors Regulate Plant Stress Responses Through Mediating Secondary Metabolism

**DOI:** 10.3390/genes11040346

**Published:** 2020-03-25

**Authors:** Tehseen Ahmad Meraj, Jingye Fu, Muhammad Ali Raza, Chenying Zhu, Qinqin Shen, Dongbei Xu, Qiang Wang

**Affiliations:** 1 Institute of Ecological Agriculture, Sichuan Agricultural University, Chengdu 611130, China; tehseenahmad55@hotmail.com (T.A.M.); fjy0204@sina.com (J.F.); cyzhu0821@163.com (C.Z.); shenqin2000@sina.cn (Q.S.); xudongbei2006@126.com (D.X.); 2 College of Agronomy, Sichuan Agricultural University, Chengdu 611130, China; Razaali0784@yahoo.com

**Keywords:** transcriptional factor, secondary metabolites, biotic stress, abiotic stress, defense

## Abstract

Plants are adapted to sense numerous stress stimuli and mount efficient defense responses by directing intricate signaling pathways. They respond to undesirable circumstances to produce stress-inducible phytochemicals that play indispensable roles in plant immunity. Extensive studies have been made to elucidate the underpinnings of defensive molecular mechanisms in various plant species. Transcriptional factors (TFs) are involved in plant defense regulations through acting as mediators by perceiving stress signals and directing downstream defense gene expression. The cross interactions of TFs and stress signaling crosstalk are decisive in determining accumulation of defense metabolites. Here, we collected the major TFs that are efficient in stress responses through regulating secondary metabolism for the direct cessation of stress factors. We focused on six major TF families including AP2/ERF, WRKY, bHLH, bZIP, MYB, and NAC. This review is the compilation of studies where researches were conducted to explore the roles of TFs in stress responses and the contribution of secondary metabolites in combating stress influences. Modulation of these TFs at transcriptional and post-transcriptional levels can facilitate molecular breeding and genetic improvement of crop plants regarding stress sensitivity and response through production of defensive compounds.

## 1. Introduction

Plants are surrounded by a myriad of diverse phytopathogens along with their sessile nature under continuously fluctuating environmental conditions. During long term evolution, plants have developed the robust and complicated innate immune systems in response to those adverse factors [1,2,3]. To deploy the natural immune mechanism, plants usually direct the growth and defense trade-off when responding to limited resources or stress conditions [4]. Regarding biotic stresses, plants mainly utilize some layers of the immune system including pathogen triggered immunity (PTI) and effector-triggered immunity (ETI) under various phases of microbial attempted infections [1,5,6]. Microbes through pathogen-associated molecular patterns (PAMPs) or microbial associated molecular patterns (MAMPs), are firstly perceived by pattern-recognition receptors (PRRs) [7]. Similarly, the abiotic stress signals are sensed by active plant sensors to initiate the complex cellular and subcellular signaling pathways as a response to environmental changes [8]. These intricate plant cellular signaling pathways are combinatory working modules that are responsible for plant behavior variation in response to prevailing stresses [3,9]. Furthermore, the complex signaling pathways against biotic and abiotic stresses involve some downstream defensive signaling such as mitogen-activated protein kinase (MAPK) cascades [10], reactive oxygen species (ROS) [11], jasmonic acid (JA) [12], salicylic acid (SA) [13], and ethylene [14,15]. These signaling pathways and their underlying crosstalk then activate transcriptional factors (TFs) that regulate plant defense through binding to the promoter regions of target genes [16].

The expression of downstream defense-related genes regulated by TFs is sometimes mediated by MAPKs [17]. The connection of MAPKs with TFs have been reviewed including TFs possessing DNA-binding structures such as ETS domain, MADS-box, zinc-finger, HMG box, bZIP domains to regulate MAPK dependent gene regulation [17]. Previously, several TFs such as HSF1, SRRA/SKN7/PRRr1, PCR1, and MYB have been associated with ROS signaling to induce a response against oxidative stress [18]. Furthermore, SA signaling has been reported extensively to rely on NPR1 and MPK4, where loss of function of these two genes resulted in the loss of pathogenesis-related (PR) genes expression [19,20,21]. In addition, the master regulator MYC2 (bHLH TF) plays a critical role in the regulation of JA signaling [22]. Other various TFs such as ORA59 belonging to the AP2/ERF family and two NAC proteins ANAC019 and ANAC055 activate downstream transcription of several JA and ethylene-responsive genes, thus involved in signaling of both phytohormones [23,24], whereas WRKY70 negatively regulated the JA responsive genes [25]. Most intriguingly, the positive or negative regulations of defense pathways are directed by complex interactions of the transcriptional protein with other proteins [26]. Taken together, defensive signal transduction leads a concerted regulation of downstream stress-responsive genes. 

In addition, pathogen recognition and downstream signaling by transcriptional activation of various defense responsive genes, the mechanisms involving the direct cessation of pathogenic colonization remain unclear. Some biological processes including cell wall reinforcement, production of anti-microbial peptides, and biosynthesis of low molecular weight secondary metabolites have been proven to be involved in the termination of infections in plants [27,28,29,30]. Plant secondary metabolites are widely suggested and deciphered compounds that contribute to plant immunity [31]. These phytochemicals include phytoanticipins and stress-inducible phytoalexins [32,33,34,35], and these antimicrobial compounds directly resist pathogenic colonization in plants [33,36]. Furthermore, these metabolites also mediate tolerance against abiotic stresses such as drought, salinity, UV irradiation, high light stresses, and ROS [37]. 

Various classes of secondary metabolites such as alkaloids, terpenoids, and flavonoids in plants have been correlated with stress responses. A number of TFs are induced by stress signals in plants, however, their roles in plant stress responses through regulating the biosynthesis of secondary metabolites have not been reviewed together. Among 58 transcription families, only a few families have been identified explicitly in plant stress responses through mediating secondary metabolism. Therefore, we reviewed a thorough perspective of plant stress responses involving secondary metabolism regulated by TFs. Following are six transcriptional families (AP2/ERF, WRKY, bHLH, bZIP, MYB, and NAC) that are involved in biotic and abiotic stress responses through mediating biosynthesis and accumulation of secondary metabolites. Owing to the space limitations, we have only covered the last five years’ researches and key references are included in this review.

## 2. AP2/ERF TFs

**Figure 1:**
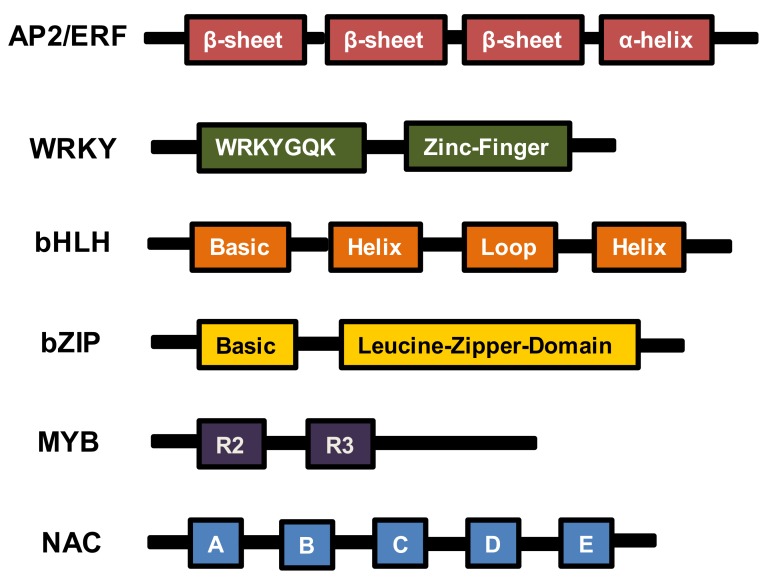
Conserved structures and domains of transcription factors in this study.

The APETALA2/Ethylene Response Factor (AP2/ERF) protein family has been studied extensively in mediating plant stress responses through involvement in the biosynthesis of secondary metabolites [38,39,40]. AP2/ERFs possess the domain of almost 60 amino acids that functionally binds to DNA sites and structurally contain three β-sheets before the α-helix motif [41] (Figure 1). This family has been classified into four subgroups, including AP2, ERF, RAV, and DREB, depending on various conserved additional domains [41,42]. 

Two AP2/ERF proteins, ORCA1 and ORCA2 were reported for the first time in *Catharanthus roseus* that bound to the promotersof terpenoid indole alkaloid (TIA) biosynthetic genes [16]. After that, a jasmonic acid-inducible ORCA3 TF was found to bind to the JERE element in promoters of two TIA biosynthesis genes encoding strictosidine synthase and tryptophan decarboxylase [43]. Recently, one AP2/ERF gene cluster containing ORCA3, 4, and 5 was identified to regulate TIA biosynthesis through mutual regulation in the cluster genes and acting on MAPKs in *C. roseus* [44,45]. These TIAs, such as catharanthine, have been related to resistance against fungi and insects by protecting the surface of *C. roseus* leaves from pathogen infections and insect infestations [46]. 

Steroidal glycoalkaloids (SGAs) defend against phytopathogens and insect infestation due to their cytotoxic properties in plants [47,48,49,50]. One TF belonging to the AP2/ERF protein family, GLYCOALKALOID METABOLISM 9 (GAME9), was reported to regulate the biosynthesis of SGAs in tobacco and *C. roseus*. Overexpression and knockdown of *GAME9* altered gene expression related to SGA production and upstream mevalonate pathway [51]. GAME9 (also named as JRE4) was bound to the promoter regions of SGA biosynthetic genes and positively regulated the gene expression of *HMGR, CAS, C5-SD, SGTs,* and *GAMEs* [52]. GAME9/JRE4 has also been reported as a primary transcriptional regulator of SGA biosynthesis in tomato against *Spodoptera litura* [47].

Nicotine, the tobacco alkaloid, has been considered as a defensive compound that facilitates plant protection from herbivores [53]. AP2/ERF genes have been reported to take part in the biosynthesis of nicotine and nornicotine in tobacco. NtERF189 and ORC1 were identified to upregulate nicotine biosynthetic gene expression and subsequent accumulation, which were also mediated by other JA-inducible factors such as NtMYC2 [54,55]. In recent years, another AP2/ERF factor NtERF32 was found to negatively regulate *NtPMT1a* gene expression, which codes putrescine N-methyltransferase to catalyze the first step of nicotine biosynthesis [56]. 

Saponins have been recognized to perform variable functions, most importantly as phytoprotectants [57,58].The AP2/ERF family has also been involved in triterpenoid saponins biosynthesis in plants. The PnERF1 of *Panax notoginseng* was identified as a positive regulator of saponins biosynthesis [59]. The binding of PnERF1 to the promoters of saponin biosynthesis genes (*HMGR, FPS, DS, SS*) increased the content of total saponins in PnERF1 overexpression lines [59]. In addition, lignin is another crucial defensive substance as abasic component of the plant cell wall. In *Gossypium barbadense*, GbERF1 was involved in lignin biosynthesis and conferred resistance against *Verticilium dahliae*. In GbERF1 overexpression and knockout lines, lignin biosynthetic genes *PAL, C4H, C3H, HCT, CoMT, CCR,* and *F5H* were upregulated and downregulated, respectively [60].

Taxol is the anticancer compoundand has also been functionally correlated with plant defense against oomycete fungi *Phytophthora capsici* [61]. Two ERF TFs, TcERF12 and TcERF15 were identified to act as the negative or positive regulator of taxol biosynthesis, respectively [62]. Both of TcERF12 and TcERF15 were bound to the *cis*-element GCC-box on the *tasy* gene promoter to regulate taxol biosynthesis. Another class of anti-microbial metabolite is hydroxycinnamic acid amide (HCAA) that confers plant defense against *Alternaria brassicicola* and *Botrytis cinerea*. The HCAA biosynthetic gene, *AtACT* (agmatinecoumaryl transferase) has been reported to be regulated by ORA59 through the bindingto two GCC boxes of *AtACT* promoter in MED25 dependent regulation [63], resulting in high accumulation of HCAAs in Arabidopsis plants. It shows that transcription factor regulation is complex and dependent on additional factors for proper accumulation of defense metabolites.

Resveratrol in grapes has been considered as an antibacterial agent that helps the plants resist against *B. cineria* [64]. Stilbene synthase (STS) is the key enzyme responsible for resveratrol biosynthesis. Most recently, VqERF114 was identified to regulate stilbene biosynthesis through binding to the *STS* promoter with another TF, VqMYB35 in *Vitis quinquangularis* [65]. In addition, ERF TF was also involved in maize defense. When attacked by lepidopteran larvae, maize leaves emit a complex blend of volatiles, mainly composed of sesquiterpenes, to attract the natural enemies of the herbivores. EREB58 is a positive regulator of sesquiterpene production by directly promoting terpene synthase *TPS10* gene expression [66]. Roles of the AP2/ERF transcriptional family have been excessively studied against biotic stresses in the past five years that clues their less involvement in abiotic stress responses (Table 1).

## 3. WRKY TFs

The WRKY transcription protein family has been studied excessively in plant defense regulations under biotic stresses. These WRKY TFs regulate gene expression through a conserved 60 amino-acid WRKY domain that precisely prevails the interaction with W-boxes of targeted gene promoters [111]. Specifically, a WRKYGQK motif locates in the N-terminus of WRKY proteins, whereas the C-terminus possesses a zinc-finger-like motif (Figure 1). The WRKY TFs are usually involved in stress responses through regulating plant secondary metabolites such as HCAAs, alkaloids, terpenoids, and other subclasses, anddiscussed in this section.

HCAAs have been revealed to functionally reinforce plant cell wall to resist the *Phytophthora infestans* colonization [67]. A potato (*Solanum tuberosum*) StWRKY1 protein was studied to be involved in HCAAs biosynthesis through regulating thephenylpropanoid pathway. StWRKY1 was directly bound to the HCAAs biosynthetic genes at their promoter regions upon infection of late blight in potatoes [67]. In wheat and barley, HCAAs were also identified to defend fungus pathogens and WRKY TFs regulated their biosynthesis. TaWRKY70 activated HCAAs related genes such as the *ACT*, *DGK*, and *GL1* to result in reduced fungal biomass on wheat plants [72]. HvWRKY23 was governing resistance against Fusarium head blight by increasing the biosynthesis of HCAAs and flavonoids through upregulating the expression of *PAL, CHS,* and *HCT* genes [76]. Moreover, the biosynthesis of resveratrol, the phytoalexin of *Vitis vinifera*, was regulated by VviWRKY24 through activating the expression of *VviSTS29*, thereby conferring antimicrobial resistance [74].

In potato, StWRKY8 positively regulated the production of benzylisoquinoline alkaloids (BIAs) through increasing the transcript levels of *TyDC, NCS,* and *COR-2* genes. BIAs as antimicrobial agents and cell wall enforcement agents were validated to eventually forbid the pathogen colonization in the late blight infestation [68]. Moreover, the maize TF ZmWRKY79 regulates the biosynthesis of phytoalexins in response to various stress conditions. Expression of several genes related to zealexins and kauralexins biosynthesis was elevated in the *ZmWRKY79* transient overexpression maize protoplasts [69]. TcWRKY1 has also been studied to play roles in taxol biosynthesis in *Taxus chinenesis* to upregulate *dbat* gene, resulting in higher accumulation of taxol [70]. Production of abietane-type diterpenoids was studied in *Salvia sclarea* to be regulated by SsWRKY18, SsWRKY40, and SsMYC2. The overexpression lines of these TFs significantly accumulated higher abietane-type diterpenoids that were responsible for resistance against various bacterial and fungal species [73]. In addition, the silencing of WsWRKY1 in *Withania somnifera* decreased phytosterol accumulation and consequently increased the susceptibility to bacteria, fungi, and insect infestation [71]. Taken together, WRKY TFs have been studied mostly in biotic stress responses through regulating secondary metabolism.

## 4. bHLH TFs

The TFs belonging to the bHLH (basic helix-loop-helix) family have been studied as potential regulators of stress-responsive mechanisms in plants. The bHLH proteins specifically comprise 60 amino-acids of bipartite conserved domains, where the basic residues at theN-terminus allow the binding of bHLH protein to DNA sites, and two alpha-helices mediate the interaction of HLH with proteins to construct homo- or heterodimeric complexes [112] (Figure 1). bHLH TFs have been characterized as efficient regulators of stress responses through regulating secondary metabolite biosynthesis, such as, flavonoids, anthocyanin, glucosinolates (GLs), diterpenoid phytoalexins, and saponins (Table 1).

bHLH TFs have been widely involved in the biosynthesis of flavonoids and anthocyanins through regulating the phenylpropanoid pathway. The master regulator of JA signaling, MYC2 belongs to the bHLH protein family [113]. Recently, MdMYC2 in apple was identified to exhibit inducible expression upon wounding and JA application, and its overexpression enhanced anthocyanin accumulation. The anthocyanin biosynthetic genes such as *DFR, UF3GT, F3H,* and *CHS* were prominently upregulated by MdMYC2 [80]. Moreover, VvbHLH1 increased accumulation of flavonoids in transgenic Arabidopsis and conferred the tolerance against drought and salinity. The expression of flavonoid biosynthesis genes *PAL, CHS, F3H,* and *DFR* was significantly upregulated in *VvbHLH1* overexpression lines [79]. 

Accumulation of anthocyaninin was also reported to be regulated by regulator complexes involving bHLH TFs. A bipartite complex through interaction between a bHLH TF, Delila, and a MYB TF, Rosea1 controlled the anthocyanin accumulation in tomato and tobacco [114]. Later, it was revealed that a tripartite MYB-bHLH-WDR (MBW) complex mediated anthocyanin and proanthocyanin biosynthesis in the Arabidopsis [115,116]. These findings suggest that the biosynthesis of plant defensive metabolites is often regulated by complexes of proteins belonging to diverse families.

Other classes of plant defensive metabolites such as GLs, diterpenoid phytoalexins, and saponins have also been reported to regulate bHLHs. A bHLH TF, IAA-LEUCINE RESISTANT3 (ILR3), played a role in the biosynthesis of aliphatic GLs that in turn conferred the resistance against nematode attack [77]. Three bHLH TFs including bHLH04, bHLH05, and bHLH06/MYC2 were also identified to regulate GLs biosynthesis through interaction with MYB51 in Arabidopsis. The triple *bhlh04/05/06* mutant lost the capability of GLs production [78]. In rice, a bHLH factor, DPF was identified as a master regulator of diterpenoid phytoalexin biosynthesis. All biosynthetic genes of diterpenoid phytoalexins exhibited higher expression in DPF overexpression lines, whereas lower expression in knockdown lines in comparison to the wild type rice plants [81]. Moreover, the susceptibility to *Gaeumannomyces graminis var. tritici* and other invaders was increased in avenacin deficient mutants of *Avena strigose* that emphasizes the role of saponins in plant resistance against invaders [57]. Recently, two bHLH TFs, TSAR1 and TSAR2 have been reported to regulate the biosynthesis of triterpene saponins through modulatingthe expression of *HMGR* gene in *Medicago truncatula* [82]. Taken together, bHLH TFs solely or interacting with other proteins regulate the biosynthesis of several secondary metabolites and play roles in both biotic and abiotic stress responses.

## 5. bZIP TFs

bZIP TFs comprise a conserved leucine zipper dimerization domain and a positively charged DNA binding interface that function in transcriptional regulation of plant biological processes (Figure 1). In plants, many secondary metabolites harboring important pharmaceutical properties such as tanshinone and artemisinin were found to be regulated by bZIP proteins including SmbZIP7, SmbZIP20 in *Salvia miltiorrhiza,* and AabZIP1 in *Artemisia annua* [117,118]. However, only a few of these medicinally important compounds were identified to be involved in plant stress response such as catharanthine [46]. 

In addition, regulating pharmaceutically important metabolites, bZIP TFs have also been extensively studied in plant defense responses through mediating biosynthesis of antipathogenic secondary metabolites. HY5, the light-responsive bZIP TF has been identified to regulate anthocyanin biosynthesis under biotic and abiotic stresses. In apple, MdHY5 is responsible for anthocyanin accumulation solely or interacting with MdMYB10 [83]. In tomato (*Solanum lycopersicum*), SlHY5 recognized the G-box and ACGT element in promoters of anthocyanin biosynthetic genes including *CHS* (chalcone synthase) and *DFR* (dihydroflavonol 4-reductase). Silencing of SlHY5 resulted in less accumulation of anthocyanin, highlighting the essential regulatory role of SlHY5 [84]. In addition, HY5 was also observed to bind to the promoter of monoterpene synthase gene *QH6* and modulated its expression [85]. 

Terpenoidphytoalexins in rice defend invasions ofthe blast pathogen and bZIP TFs also regulate their biosynthesis besides the bHLH TF, DPF as mentioned above. The bZIP TF, OsTGAP1 plays the important rolein regulating of rice terpenoid phytoalexin biosynthesis through binding to the promoters of *OsKSL4* and *OsCPS4*, two essential terpene synthase encoding genes. Overexpression of *OsTGAP1* also enhanced expression of other terpenoid biosynthesis genes and elevated the upstream MEP pathway that provides the isoprenoid precursors for terpenoid phytoalexin biosynthesis [86,87,88]. In contrast, OsbZIP79 played as a negative regulator of terpenoid phytoalexin biosynthesis in rice. OsbZIP79 overexpression lines showed downregulation for phytoalexin biosynthetic genes and some MEP pathway genes such as *DXS*, with suppressed phytoalexin accumulation [89].

## 6. MYB TFs

MYB TFs are widely involved in plant biological processes including growth, reproduction, and stress responses. The MYB TF family is divided into four subgroups, 1R, 2R, 3R, and 4R, depending on the repeats of DNA binding domains. Each domain contains 50–53 amino acids that form α-helices, whereas a helix-turn-helix is located between the second and third helices that facilitates MYB TFs binding to target DNA sequences [119] (Figure 1).

Many secondary metabolites have also been reported to be mediated by MYB proteins, such as GLs, flavonoids, HCAAs, and proanthocynanins. Indole glucosinolates (IGs) have been suggested as defensive compounds especially in the case of biotic stress in plants [120]. In Arabidopsis, MYB34, MYB51, and MYB122 distinctly took part in tryptophan-derived IGs biosynthesis by elevating the gene expression of two cytochrome P450s, CYP79B2, and CYP79B3 that catalyze the initial step of IG biosynthesis [90]. Further, the *myb34/51/122* triple mutant exhibited significantly reduced IGs production and increased susceptibility to *Plectosphaerella cucumerina* that substantially proved the role of these MYBTFs in disease resistance through regulating IG metabolism [91]. 

Regarding flavonoid biosynthesis, a citrus MYB factor, CsMYBF1, positively regulates flavanol production through acting on the *CHS* gene [96]. In addition, SbMYB8, a TF from Chinese medical herb *Scutellaria baicalensis*, regulated *CHS* gene expression and increased flavonoid accumulation in transgenic tobacco, conferring elevated tolerance against drought stress [103]. Recently, flavonoids in tea (*Camellia sinensis*) were identified to mediate resistance against a crop destructive disease blister blight caused by *Exobasidium vaxans* [121]. The flavonoid biosynthesis in tea plants was regulated by CsMYB2/CsMYB26 that was directly bound to the promoters of *CsF3’H* and *CsLAR*, two flavonoid biosynthetic genes [104]. These two MYB TFs exhibited correlated inducible gene expression with their target genes upon pathogen infection [104]. Moreover, three MYB TFs, AtMYB11, AtMYB12, and AtMYB111 were revealed to differentially regulate the flavonoid biosynthesis [97,98,99]. Heterologous overexpression of these AtMYBs resulted in flavonoid accumulation in various plant species [100,101], where the alteration of flavonoid metabolism subsequently modulated stress tolerance [122]. MYB TFs were also identified to play negative roles in flavonoid metabolism. Overexpression of AtMYB75 in Arabidopsis reduced the level of kaempferol-3,7-dirhamnoside. It resulted in increased susceptibility to insect herbivore *Pieris brassicae*, whereas growth of caterpillar was reduced significantly upon exogenous application of kaempferol-3,7-dirhamnoside [92].

In rice, HCAAs conferred resistance against invaders by regulation of three MYB TFs, MYB30, MYB55, and MYB110 [105]. These MYB TFs acted on phenylpropanoid biosynthetic genes such as *HCT*, *4CL3*, *C4H,* and *PAL* to regulate HCCAs accumulation [105]. MYB TFs also regulate proanthocyanins biosynthesis. Recently, two proanthocyanin biosynthesis genes *ANR1* and *LAR3* in poplar have been observed to be upregulated in PtMYB115 overexpression lines, resulting in increased proanthocyanin accumulation. High accumulation of proanthocyanin in PtMYB115-OE lines subsequently enhanced the resistance against fungal pathogen *Dothiorella gregaria*, whereas the *Ptmyb115* mutant exhibited susceptibility to this pathogen [93].

In contrast, the grape MYB TF, VvMYBC2-L1 negatively regulated proanthocyanin biosynthesis by downregulating the corresponding biosynthesis genes, *VvDFR*, *VvLDOX*, *VvLAR1/VvLAR2*, and *VvANR* [94]. Moreover, accumulation of proanthocyanins also elevated the resistance against wounding and oxidative stress in *Rosa rugosa*, which was regulated by RrMYB5 and RrMYB10. Two proanthocyanin biosynthetic genes, *RrANR* and *RrDFR* were consequently upregulated or downregulated in *RrMYB5/RrMYB10* overexpression or knockout *R.rugosa* lines, respectively [95]. 

## 7. NAC TFs

The transcriptional proteins belonging NAC family have been widely recognized as plant biotic and abiotic stress-responsive factors [123,124,125]. The word NAC is given upon previously characterized proteins from Petunia NAM (nonapical meristem), and Arabidopsis ATAF1/2 and CUC2 (cup-shaped cotyledon) that possessed the conserved NAC domains [125,126]. The NAC family contains a DNA binding domain at the N-terminus and a conserved activation domain at the C-terminus (Figure 1). Many NAC TFs have been studied and identified to play roles in biotic and abiotic stress responses. However, only a few cases are reported about the molecular mechanism in stress response through mediating plant secondary metabolism. 

PtrNAC72 from *Poncirus trifoliate* was reported to regulate putrescine biosynthesis by controlling the gene expression of arginine decarboxylase (*ADC*), subsequently mediating ROS homeostasis [106]. The transgenic Arabidopsis plants with PtrNAC72 overexpression accumulated relatively low putrescine through downregulating *ADC* gene expression, while higher putrescine accumulation was detected in T-DNA inserted mutant *nac72*, resulting in decreased and increased drought resistance, respectively [106]. In addition, glutathione is a prototypic anti-oxidative metabolite that functionally eliminates the harmful effects of drought produced ROS [127]. MfNACsa in *Medicago falcate* was responsive for defending drought stress by regulating the gene expression of *glyoxalase 1* to maintain glutathione level [110].

In plants, flavonoids are widely involved in stress response [128]. Flavonoids accumulation in Norway spruce (*Picea abies*) increased the resistance against the fungal pathogen *Heterobasidion annosum* [129]). Recently, it was revealed that PaNAC03 responded against infection of *H. annosum* by negatively regulating some flavonoid biosynthetic genes, such as *CHS*, *F3’H,* and *LAR3* [107]. Moreover, ANACO32 has also been identified as a negative regulator of anthocyanin biosynthesis. The overexpression lines of ANACO32 exhibited downregulation of some anthocyanin biosynthetic genes including *DFR*, *ANS,* and *LODX* under diverse stress conditions [108]. 

In *Hevea brasiliensis*, latex biosynthesis has been commonly induced by tapping and wounding strategies for rubber production, which might be correlated with different stress signals such as dehydration, ROS, and jasmonates [130]. A *H. brasiliensis* NAC TF, HbNAC1 was identified to be involved in latex biosynthesis and drought tolerance. The dehydration-induced HbNAC1 was directly bound to the CACG motif in the promoter of *SRPP* (small rubber particle protein) to regulate latex biosynthesis [109]. 

## 8. Cross Interactions of Diverse Transcription Proteins Show the Complexity of Phytoprotective Regulation

Plant proteins usually work in homo- or heteromutimeric complexes to regulate stress responses [116]. Multiple signals are involved in regulation of the secondary metabolism, as well as plant stress responses, which reasonably requires complicated signaling crosstalk and regulator interaction. In the context of TFs interactions, several heteromutimeric protein complexes are involved in stress response through mediating secondary metabolism, such as the MYB-bHLH-WDR complex in Arabidopsis that plays the crucial role for the biosynthesis of flavonoids [116]. Such regulation is suppressed by the interaction of JAZs and MYBL2 protein. However, this suppression can be overcome by DELLA proteins that sequester JAZs and MYBL2 to make an interaction with the MBW complex [131]. In Norway spruce, another MYB-bHLH-WDR complex also regulates flavonoid biosynthesis [115]. GLs biosynthesis also undergoes multiple regulations through the physical interaction of MYBs and bHLHs [26,78]. GAME9 acts on SGA biosynthesis through interacting with MYC2, the master regulation of JA signaling [51].

*STS*, the key biosynthetic gene of resveraterol, is regulated by MYB TFs through multiple interactions with other factors. VviMYB14 interacted with VviWRKY03 in *V. vinifera* to upregulate the *STS* gene [74]. In Chinese wild grape *V. quinquangularis*, a similar mechanism was observed for VqERF114 and VqMYB35 that positively regulated *STS* gene expression through physical protein interaction [65]. In contrast, VvWRKY8 physically interacted with VvMYB14 and prevented its binding on *STS* gene promoter and subsequently repressed *STS* gene expression and resveraterol synthesis [75]. 

**Figure 2:**
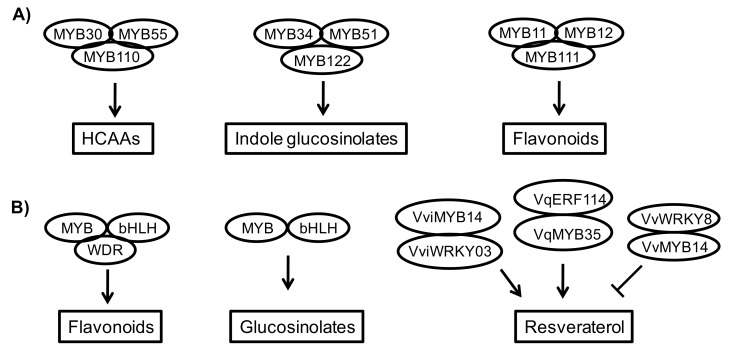
Protein interaction of transcription factors in the regulation of secondary metabolism. Homomutimeric complexes (**A**) and heteromutimeric complexes (**B**) of transcription factors regulate the biosynthesis of diverse secondary metabolites through protein interaction.

Some MYB TFs also form homomutimeric complexes to regulate the biosynthesis of IGs, HCAAs, and flavonoids in plants including the MYB34-MYB51-MYB122 complex, the MYB30-MYB55-MYB110 complex, and the MYB11-MYB12-MYB111 complex, respectively [78,90,97,98,99,105] (Figure 2). 

## 9. Concluding Remarks and Future Perspectives

**Figure 3:**
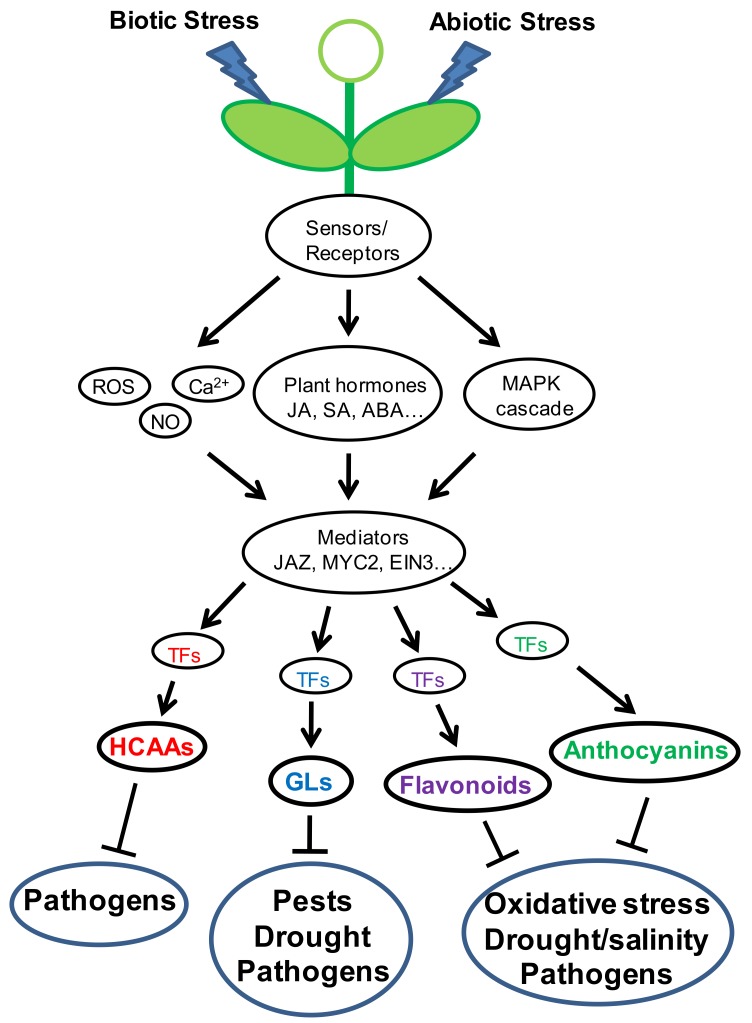
Signal transduction and regulation of secondary metabolism in response to biotic/abiotic stresses in Arabidopsis. Four types of secondary metabolites in Arabidopsis involved in different resistances are exemplified to show the regulation of secondary metabolism by different transcription factors (TFs), which are mediated by the complicated upstream signaling pathways in response to stresses. The TFs and their corresponding regulated metabolites are labeled with the same colors.

Plants are usually exposed to multiple stresses simultaneously or continually. Cross resistance is widely observed in plants and has been utilized in crop cultivation. In response to these diverse biotic/abiotic stresses, plants perceive these adverse cues through different sensors/receptors, which undergo structure transformation/modification upon acceptance of exogenous ligands, and then transfer signals to downstream processes. Many biological molecules and processes are induced/initiated such as plant hormones, ROS, Ca^2+^, and MAPK cascade. Signals are further transduced by a number of mediators such as JAZ, MYC2, EIN3 to the downstream TFs. These TFs directly bind to the *cis*-elements on the gene promoters to regulate gene expression and subsequent biosynthesis of secondary metabolites. Some secondary metabolites may directly inhibit pathogen infection or pest infestation. Others might participate into ROS scavenging to keep redox balance and confer drought/salinity tolerance to plants. Signaling crosstalk is observed in the whole pathway of signal transduction. Different stress factors might converge to activate the same signaling pathway and induce biosynthesis of the same secondary metabolites. Some secondary metabolites are also involved in resistance/tolerance against different stresses (Figure 3). For example, GLs usually exhibit toxicity to pathogens and insect herbivores. It was also reported to regulate stomatal movement and be involved in drought resistance [132]. Additionally, maize terpenoid phytoalexins, kauralexins defend fungal pathogens and exhibit antifeedant activity against corn borer [133]. However, the kauralexin-deficient *an2* mutant is susceptible to drought stress in comparison to the wild type W22 plant, implicating involvement of kauralexins in abiotic stress responses [134]. Hence, more investigation should be conducted to explore the complicated regulation of secondary metabolism involved in stress response.

To date, thousands of constitutive and stress-inducible secondary metabolites conferring plant tolerance and resistance have been reported in diverse plant species, only a small part of these metabolites has been discussed in the present review. Most importantly, the optimum accumulation of stress-responsive metabolites is beneficial for crop productivity and resistance against both biotic and abiotic stresses. The higher accumulation of plant defensive metabolites may create toxicity and compromise on the quality of plant products. On the other side, the low accumulation may influence plant resistance or tolerance against stress conditions. The optimum accumulation would make a balance between plant growth and defense which may stabilize the ultimate plant yield by sustaining plant growth with defense against invasions.

Transcriptional factors can be utilized in molecular breeding to enhance the plant yield by optimizing the level of metabolite flux. However, a single transcription factor is often insufficient to regulate the whole biosynthetic pathway of secondary metabolite, which in most time needs the regulatory networks consisting of multiple TFs. Co-expression analysis regarding protein–protein interactions (interactomics) and investigations regardingprotein nucleocytoplasmic partition or subcellular trafficking are crucial to explore the underlying mechanisms of these TFs mediating secondary metabolism to respond diverse stresses. These researches may enhance the knowledge of plant metabolism and would be helpful to modify the genetic architecture to get highly adaptable crop plant varieties. 

This review emphasizes the plant sensitivity and responsiveness against unfavorable conditions and underlying regulatory mechanisms. Here, the roles of plant TFs and secondary metabolites in diverse stress conditions have been studied together. In future, studies of TFs based mechanisms and regulations regarding stress can proceed to elucidate the responsible metabolites that are involved in the direct cessation of stress factors. Further, the modulation of regulatory machine for optimized metabolite biosynthesis and accumulation would be helpful for molecular breeding of crop plants to increase the sensitivity and responsiveness under sustainable growth conditions. 

## Figures and Tables

**Table 1:** Plant TFs mediated stress response through regulating secondary metabolite biosynthesis.

Families	Transcriptional Factors	Plant Species	Metabolites	Resistance	References
AP2/ERF	JRE4 (GAME9)	*S. lycopersicum, S. tuberosum*	SGAs	*S. litura*	[47,51]
	NtERF32	*N. tabaccum*	Nicotine	Toxic against herbivory	[56]
	GbERF1	*G. barbadense*	Lignin	*V. dahliae*	[60]
	ORA59	*A. thaliana*	HCAAs	*A. brassicicola, B. cineria*	[63]
	TcERF12/ TcERF15	*T. chinensis*	Taxol	*P. capsici*	[62]
	VqERF114	*V. quinquangularis*	Resveratrol	*B. cineria*	[65]
	EREB58	*Z. mays*	Sesquiterpenes	Defence against herbivory	[66]
	PnERF1	*P. notoginseng*	Saponins	Anti-microbial	[59]
WRKY	StWRKY1	*S. tuberosum*	HCAAs	Anti-microbial	[67]
	StWRKY8	*S. tuberosum*	BIAs	*P. infestance*	[68]
	ZmWRKY79	*Z. mays*	Terpenoid phytoalexins	Anti-microbial	[69]
	TcWRKY1	*T. chinenesis*	Taxol	Anti-microbial	[70]
	WsWRKY1	*W. somnifera*	Phytosterol	Bacteria, Fungi and Insect	[71]
	TaWRKY70	*T. aestivum*	HCAAs	Fungi	[72]
	SsWRKY18/ 40	*S. sclarea*	Diterpenoids	Bacteria and Fungi	[73]
	VviWRKY24/03/VvWRKY8	*V. vinifera*	Resveratrol	*B. cineria*	[74,75]
	HvWRKY23	*H. vulgare*	HCAAs	Fusarium head blight	[76]
bHLH	ILR3/bHLH104, bHLH04/05/06	*A. thaliana*	GLs	*H. schachtii*	[77,78]
	VvbHLH1	*A. thaliana*	Flavoniods	Drought and Salt	[79]
	MdMYC2	*M. domestica*	Anthocyanin	Anti-pathogenic, Drought and Salinity	[80]
	DPF	*O. sativa*	Diterpenoid phytoalexins	Anti-Pathogenic	[81]
	TSAR1/TSAR2	*M. falcata*	Saponins	Anti-microbial	[82]
bZIP	MdHY5	*M. domestica*	Anthocyanin	Drought, Pathogen and Salinity	[83]
	SlHY5	*S. lycopersicum*	AnthocyaninMonoterpenoids	Drought, Pathogen and SalinityAnti-pathogenic	[84,85]
	OsTGAP1	*O. sativa*	Diterpenoid phytoalexins	Anti-pathogenic	[86,87,88]
	OsbZIP79	*O. sativa*	Diterpenoid phytoalexins	Anti-pathogenic	[89]
MYB	AtMYB34/51/112	*A. thaliana*	IGS	*Plectospharella cucumerina*	[90,91]
	AtMYB75	*A. thaliana*	Anthocyanin	*Pieris brassicae*	[92]
	PtMYB115	*P. tomentosa*	Proanthocyanin	*Dothiorella gregaria*	[93]
	VvMYBC2-L1	*V. vinifera*	Proanthocyanin	Wounding and Oxidative stress	[94]
	VvMYB14/VviMYB14	*V. vinifera*	Resveratrol	*B. cineria*	[74,75]
	RrMYB5/ RrMYB10	*R. rugosa*	Proanthocyanin	Wounding and Oxidative stress	[95]
	CsMYBF1	*Citrus sinensis*	Flavonoids and HCAAs	Antimicrobial	[96]
	AtMYB11/12/111	*A. thaliana*	Flavonoids	Antimicrobial, Salinity	[97,98,99,100,101,102]
	SbMYB8	*S. baicalensis*	Flavonoids	Drought	[103]
	CsMYB2/26	*C. sinensis*	Flavonoids	Blister Blight	[104]
	OsMYB30/55/110	*O. sativa*	HCAAs	Fungi and Bacteria	[105]
NAC	PtrNAC72	*P. trifoliata*	Putriscene	Drought	[106]
	PaNAC03	*P. abies*	Flavonoid	*H. annosum*	[107]
	ANACO32	*A. thaliana*	Anthocyanin	Drought and Salinity	[108]
	HbNAC1	*H. brasiliensis*	Latex	Drought	[109]
	MfNACsa	*M. falcata*	Glutathione	Drought	[110]
